# Patient and provider acceptance of telecoaching in type 2 diabetes: a mixed-method study embedded in a randomised clinical trial

**DOI:** 10.1186/s12911-016-0383-3

**Published:** 2016-11-09

**Authors:** I. Odnoletkova, H. Buysse, F. Nobels, G. Goderis, B. Aertgeerts, L. Annemans, D. Ramaekers

**Affiliations:** 1 Leuven Institute for Healthcare Policy, KU Leuven, Kapucijnenvoer 33, Leuven, B-3000 Belgium; 2 Department of Public Health, Ghent University, De Pintelaan 185, 4K3, 9000 Ghent, Belgium; 3 Department of Endocrinology, OLV Hospital Aalst, Moorselbaan 164, 9300 Aalst, Belgium; 4 Academic Center for General Practice, KU Leuven, Kapucijnenvoer 33, Leuven, B-3000 Belgium

**Keywords:** Patient education, Telecare, Type 2 diabetes, Qualitative research

## Abstract

**Background:**

Despite advances in diagnosis and treatment of type 2 diabetes, suboptimal metabolic control persists. Patient education in diabetes has been proved to enhance self-efficacy and guideline-driven treatment, however many people with type 2 diabetes do not have access to or do not participate in self-management support programmes. Tele-education and telecoaching have the potential to improve accessibility and efficiency of care, but there is a slow uptake in Europe. Patient and provider acceptance in a local context is an important pre-condition for implementation. The aim of the study was to explore the perceptions of patients, nurses and general practitioners (GPs) regarding telecoaching in type 2 diabetes.

**Methods:**

Mixed-method study embedded in a clinical trial, in which a nurse-led target-driven telecoaching programme consisting of 5 monthly telephone sessions of +/− 30 min was offered to 287 people with type 2 diabetes in Belgian primary care. Intervention attendance and satisfaction about the programme were analysed along with qualitative data obtained during post-trial semi-structured interviews with a purposive sample of patients, general practitioners (GPs) and nurses. The perceptions of patients and care providers about the intervention were coded and the themes interpreted as barriers or facilitators for adoption.

**Results:**

Of 252 patients available for a follow-up analysis, 97.5 % reported being satisfied. Interviews were held with 16 patients, 17 general practitioners (GPs) and all nurses involved (*n* = 6). Themes associated with adoption facilitation were: 1) improved diabetes control; 2) need for more tailored patient education programmes offered from the moment of diagnosis; 3) comfort and flexibility; 4) evidence-based nature of the programme; 5) established cooperation between GPs and diabetes educators; and 6) efficiency gains. Most potential barriers were derived from the provider views: 1) poor patient motivation and suboptimal compliance with “faceless” advice; 2) GPs’ reluctance in the area of patient referral and information sharing; 3) lack of legal, organisational and financial framework for telecare.

**Conclusions:**

Nurse-led telecoaching of people with type 2 diabetes was well-accepted by patients and providers, with providers being in general more critical in their reflections. With increasing patient demand for mobile and remote services in healthcare, the findings of this study should support professionals involved in healthcare policy and innovation.

**Trial registration:**

NCT01612520, registered prior to recruitment on 4th June 2012.

**Electronic supplementary material:**

The online version of this article (doi:10.1186/s12911-016-0383-3) contains supplementary material, which is available to authorized users.

## Background

About 415 million people worldwide have diabetes and its prevalence is expected to increase by more than 50 % in the coming 20 years [1]. Despite significant advances in diagnosis and treatment, inadequate metabolic control persists. Poor risk factor control may be reflected by both the failure of diabetes self-management by patients as well as inadequate intervention strategies by clinicians [2]. Patient education has been proved to enhance self-care and guidelines-driven diabetes treatment [3–5].

In Belgium, patient education was initially introduced in 1988 for people with advanced diabetes in a hospital setting and extended to primary care in 2009, where it has been delivered by certified diabetes educators, mostly in individual sessions at the patient’s home. Within the current delivery model, a significant amount of nurses’ time and budget (~50 % of total) is dedicated to transport and administration. Diabetes education is not reimbursed to patients in the early stage of diabetes – those on lifestyle and/or oral antidiabetic therapy. Alternative more efficient approaches are needed to ensure better patient inclusion.

Advances in internet-based technologies offer a variety of tools for efficient video- and audio communication and information exchange to support distant patient self-management support. Overall, internet penetration in Western countries has reached 85 %, ensuring basic infrastructure for such services [6]. However, among people above 65 years old, the age group where such support is especially needed, internet use is usually lower and does not exceed 60 % in Belgium [7]. For this age group, the telephone still represents a practical solution for communication with healthcare professionals and has the potential to improve the quality and accessibility of chronic care [8, 9].

Distant care solutions have had a slow uptake in Europe [10, 11]. The adoption of evidence-based patient support interventions in daily clinical practice depends on the social and organisational context in which they are introduced and used [2]. This paper explores the patient and provider perceptions about diabetes education by phone in Belgian primary care setting. The study participants were involved in a randomised clinical trial (RCT) which was the first to test a nurse-led telecoaching programme in Belgium and showed sustainable improvement in glycaemic control [12].

The objective of this research was to explore the perceptions of patients, nurses and general practitioners (GPs) involved in the RCT, regarding the barriers and facilitators to implementation and adoption of nurse-led telecoaching in Belgian primary care.

## Methods

### Intervention and setting

Between April 2012 and January 2014, 3115 people on glycaemia-lowering agents were invited to participate in the study by the Independent Health Insurance Fund of Belgium. Two hundred and eighty-seven patients with type 2 diabetes were randomised to the intervention group and received the COACH Program (TCP), delivered by certified diabetes nurse educators after additional training [13]. The intervention consisted of 5 monthly telephone sessions of 30 min on average and was focused on achieving guideline-recommended diabetes treatment targets through regular control of diabetes risk factors including self-monitoring of blood glucose, appropriate lifestyle adjustments and intensification of medication therapy upon a patient consultation with GP [12].

The novel features of TCP compared to usual diabetes education were: 1) focus on closing the “treatment gaps”, i.e. failure to achieve the guideline recommended goals, by systematically covering each diabetes risk factor based on the clinical guidelines; 2) analysing patient risk profile based on recent lab results and a standard intake interview; 3) delivering the coaching entirely by phone; 4) sharing a written patient progress report with the patient and his GP; 5) using special software for patient administration.

### Research design

A programme satisfaction questionnaire was filled out during a nurse home visit upon graduation from TCP, by all patients who were available for the RCT follow-up assessment. The survey was a five-point Likert scale questionnaire developed for TCP evaluation (Table 1) [13]. It was translated into Dutch and piloted for intelligibility with several patients. Patient attrition and associated reasons were registered by nurses who delivered the intervention.

**Table 1:** Results of the questionnaire about patient satisfaction with the COACH Program, based on the responses of 252 participants of the intervention group

Score	5	4	3	2	1
Questionnaire dimension	Strongly agree				Strongly disagree
The telephone was an effective form of communication between me and my coach.	70.5 %	21.6 %	6.2 %	1.7 %	0 %
I feel better informed about my diabetes risk factors than before I joined The COACH Program.	68.5 %	24.5 %	5.8 %	1.2 %	0 %
The coach listened to me and gave advice that was relevant to my needs.	78.3 %	17.5 %	3.8 %	0.4 %	0 %
The written progress reports were useful.	69.3 %	21.6 %	7.9 %	0.4 %	0.8 %
Sending a copy of the progress reports to my treating physician was useful.	72.4 %	15.9 %	7.1 %	2.9 %	1.7 %
The time interval between coaching sessions was appropriate.	74.5 %	19.2	5.4 %	0.4 %	0.4 %
Overall, I was satisfied with The COACH Program.	78.4 %	19.1 %	1.7 %	0.4 %	0.4 %

Semi-structured individual face-to face interviews were held with the trial participants: all nurses and a purposive sample of patients and GPs. Patients and GPs were randomly selected from the trial participant list and invited for a phone interview with a trial assistant. The respondents were recruited and interviewed until a theoretical saturation point was achieved, i.e. no new themes could be derived from additional interviews.

### The interviews

The interviews were designed and performed by IO and HB. A flexible topic guide was used (Additional file 1). Challenges in diabetes management, perceptions about telecounselling and the COACH Program were consistently discussed with all respondents. The purpose of the interviews was to let the respondent talk freely about a subject in order to be able to capture as much information as possible. To encourage a discussion, “reflective listening” techniques were applied [14]. HB and IO agreed on the conduct of the interviews and used specific tutorials in the preparation phase [15]. The interviews were piloted with one respondent from each target group. All conversations were audio-taped and transcribed verbatim.

### Data analysis

To process the qualitative data, consensual analytic techniques were used [16–19]. NVivo 10 software was employed to assist the data coding. Directed content analysis was applied, i.e. a priory developed topic guide was used as the initial coding scheme. The analysis was performed in four stages: 1) interview transcription; 2) assigning quotes to the discussion topics; 3) compressing quotes and extracting the content core (themes); 4) interpreting themes associated with barriers or facilitators for adoption. A multiple coding approach was used, i.e. IO and HB performed the coding exercise and cross-revised the transcriptions and interpretation of data. Upon a consensus, the results were reviewed by and discussed within the research team.

To classify the identified themes based on their relevance for implementation and adoption of telecoaching, the “menu” of constructs of the Consolidated Framework for Implementation Research (CFIR) was used [20]. Suitable for a variety of research purposes, this comprehensive framework is recommended as a practical guide for systematically assessing potential barriers and facilitators in preparation for implementing an innovation. Three domains were selected based on their relevance and supported by applicable constructs. These include: Intervention characteristics (Benefit for patient and Key features of the intervention); Implementation process (Engaging of GPs & Patient recruitment; Executing & Quality assurance); and Broader context (Current practice of patient education in type 2 diabetes and Personal beliefs about telecounselling). The themes associated with barriers and facilitators are discussed per respondent group and supported by the most representative quotes.

The intervention attendance rate and the COACH Program satisfaction questionnaire results were analysed with IBM SPSS Statistics 22 software.

## Results

### Intervention fidelity and patient satisfaction

Of 287 patients enrolled in the telecoaching group, 252 (87.8 %) were available for the RCT follow-up assessment. Patients reported a high level of satisfaction with The COACH Program in general (97.5 %). 92.1 % were content with the telephone as a medium for communicating with the coach (Table 1).

### Interview participants

Between April and July 2014, 6 nurses, 16 patients and 17 GPs were interviewed (Table 2). Twenty-nine percent of initially contacted patients and 57 % of GPs refused to participate in the study, with lack of time as the most frequently reported reason.

**Table 2:** Characteristics of the interview participants

	N	Men, n (%)	Age, median (min-max)	Years, median (min-max)
Patients	16	7 (44 %)	68 (37–77)	11 (3–20)^a^
GPs	17	12 (71 %)	51 (33–69)	25 (7–46)^b^
Nurses	6	2 (33 %)	37 (32–51)	5 (3–11)^c^

^a^with diagnosis type 2 diabetes

^b^GP experience (65 % working in a solo practice)

^c^experience as diabetes educator

### Interview results

Themes associated with the barriers and facilitators for implementation of nurse-led telecoaching in type 2 diabetes in Belgian primary care are summarised below per respondent group and in Additional file 2: Table S1 per CFIR domain applied.

#### Potential facilitators for adoption

##### Views shared by patients, nurses and GPs

Overall, the respondents found TCP to be a benefit for patients and mentioned at least one of the following improvements: understanding of diabetes, motivation, discipline in diet habits and physical activity, more regular check-ups and better risk factor control. There was a general acknowledgment of the fact that GPs don’t have enough time to provide diabetes education. Most interviewees seemed to be convinced of the importance of diabetes education from the moment of the diagnosis and emphasised the need for a variety of education programmes tailored to patient needs. Inequalities in the reimbursement of diabetes education were criticised by most.

*Nurse 1: “Diabetes education should start from the moment of the diagnosis and maybe even earlier, in the prediabetes stage. The patients must be directly informed how they can self-manage diabetes, in the first place through adjustments in the eating habits, physical activity. It is essential that the patient understands the possible complications, so he/she can react more quickly to certain symptoms. Apparently, today there are not enough resources for that.”*

The majority of respondents believed that telecounselling can help to achieve an efficiency gain and better care accessibility through partial substitution of face-to-face contacts.

##### Perception of patients

Patients reported being satisfied with TCP. Regular repetition and monitoring by the coach was perceived by most as an important vehicle for lifestyle change.

*Patient 15 “I started to pay more attention to my diet. You know that they’re going to call and it is like a big stick, − you try to do your best. I lost several kilos and am glad about it… I have learned a lot about self-monitoring. Diet advice and glucose monitoring were the most important things.”*

Patients found communication by phone comfortable, time-saving and flexible.

*Patient 1: “You don’t need to travel and yet you can ask questions and directly receive an answer… I have to visit the hospital regularly because of other problems. That’s why I think: Let me for once stay at home.”*

About half of the interviewed patients found that the programme should not have stopped after 6 months, but continued at lower intensity, or re-launched on the occasion of a treatment adjustment, such as initiation of insulin therapy.

##### Perceptions of GPs

Based on the experience of cooperation with diabetes educators in previous years, the majority of GPs expressed trust in their performance. They thought that the educators facilitated their work.

*GP 5 “Diabetes educators are a great added value. It should have been introduced a long time ago. What a patient needs to know (self-injections, diet) and where we lack time or knowledge, they come in-between.”*

Most found information about the launch of TCP to be sufficient. The fact that the programme did not require much additional time investment, was valued by GPs. GPs were in general satisfied about the quality of the advice within the written patient progress reports. Most of them have integrated the electronic copies of the progress reports into the patient medical records. All but one GP thought that motivated nurse advice on therapy adjustment was acceptable.

##### Perception of nurses

All nurses thought that the current concept of diabetes education needs to be revised. They complained about underfinancing due to high transport costs.

*Nurse 3: “We have to travel from patient to patient and these costs are barely covered. Ideally, I would start the education with a home visit and do the rest via the telephone or via internet, if possible.”*

All found that TCP and associated software offer a clear structure. The 1-week training course in individual risk factor targets and medication management was perceived as knowledge advancement.

*Nurse 6: “Looking back at the training, I see that I have grown in my profession. The training covered the medication therapy and when/how it should be adjusted. I use this knowledge in my work outside of The COACH Program as well.”*

Discussing with patients the blood glucose levels based on the self-monitoring results was considered by all as important guidance for appropriate advice. All nurses mentioned that observing improvements in patient diabetes control was rewarding. They appreciated receiving the results of patient baseline clinical assessments at the start of the programme as well as the follow-up results. Some admitted that comprehensive patient information is frequently absent from their usual education practice.

#### Potential barriers for adoption

##### Views shared by patients, nurses and GPs

Lack of discipline and commitment, particularly in lifestyle recommendations, was identified by most interviewees as the major challenge in leveraging the effect of diabetes treatment in general and educational programmes in particular.

*GP 17: “Motivation of the patient is the biggest concern. I keep harping on, but the patients do not appreciate it, some totally ignore my advice… The problem is: diabetes does not hurt. It starts hurting when it is already too late…”*

##### Perceptions of patients

Overall, patients found it challenging to remember and understand the targets for all risk factors associated with diabetes. The patient who was not satisfied with TCP, mentioned that he dislikes communicating by phone in general and that he participated in the programme just to please his wife.

##### Perceptions of GPs

Some GPs admitted being reluctant about the programme in the beginning, because they are overwhelmed with initiatives and have difficulty keeping track of the source, goals and quality of different pilot projects.

Several GPs regretted that the patient progress reports were not sent electronically for direct integration into the patient medical record. Some felt there should have been direct interaction with the coach, particularly about appropriate therapy adjustment. One GP had a negative perception about diabetes education in general because of a limited availability of diabetes educators. Several complained about the administrative burden associated with the current referral procedure.

According to most GPs, work still needs to be done to identify the groups of patients who would benefit from consultations or coaching by phone. Some expressed concerns about inclusion of older patients because of fading perceptive capabilities. All GPs thought that such interventions could not entirely substitute a personal contact and emphasised the current lack of a legal, financial and organisational framework.

*GP 7 “Telephone support is perfectly possible. My patients who are abroad, call me regularly for a consultation. For me, this is voluntary work, because currently I cannot make an invoice for a tele-consultation. This should not be difficult to organise because many people are open to this…”*

In addition, most GPs thought that the current fee-for-service payment system is a poor fit with chronic and multidisciplinary care.

##### Perceptions of nurses

While recognising certain benefits of telecoaching, the nurses also brought up some limitations. Diabetes education entirely by phone was perceived as a barrier by most nurses. All would have preferred starting the programme with a face-to-face visit to the patient. They thought that such contact would create more trust and increase patient commitment. Moreover, meeting the patient at home would have provided important information on the patient living environment and lifestyle, which they did not currently have.

Nurse 2 *“I thought it was not easy to do the entire programme by phone… I think, if they had once met me personally, they would have sometimes made more effort. For the future, I would see a combination of a home visit and the telephone sessions as the ideal solution.”*

The nurses were not used to making extensive patient progress reports with specific advice and were sometimes uncertain about the acceptance of their recommendations by GPs. Overall, they found GPs reluctant to up-titrate medications, even when they thought it was necessary. They also complained about the attitude of patients who postpone visiting their GPs.

In the beginning, the coaches found it difficult to adopt a new way of working. Getting used to the TCP software and preparing the progress reports was experienced as time-consuming. Sometimes technical issues, time pressure due to other regular nursing tasks, and the occasional failure of patients to keep their appointment caused frustrations among the nurses. However, their attitude towards the programme improved as they gained more experience.

*Nurse 4: “Making reports was time-consuming. We are not used to making these kinds of detailed reports. On the other hand, it was a good thing that both the patient and the GP received them. This reinforced the responsibility of the patient.”*

In general, diabetes educators think that there is room for improvement in their cooperation with GPs. They criticise the lack of readiness for cooperation and information exchange among some GPs and their selective referral behaviour.

*Nurse 2: “Communication with GPs – we invest a lot of time in it… First, they are not easy to reach. If you reach them, they do not take time to look up the information we need. But there are GPs who are more cooperative, I have the feeling that it’s improving… You need to be very patient with them.”*

## Discussion

This study investigated the acceptance of a clinically effective nurse-led telecoaching programme in type 2 diabetes among the participants of the clinical trial, − patients, nurses and GPs. The main objective of telecoaching was to make the patient aware of the individual risk factor targets associated with diabetes and empower the patient to take responsibility for achieving and maintaining these individual targets. The randomised controlled trial in a Belgian primary care setting demonstrated that even though, at baseline the study population was already quite well controlled for most diabetes risk factors, telecoaching resulted in a clinically modest HbA1c reduction by 2 mmol/mol (0.2 %) in the total sample and a clinically significant reduction by 4 mmol/mol (0.4 %) in the subgroup of patients with HbA1c ≥ 53 mmol/mol (7 %) at baseline. These improvements in glycaemic control were still observed at 18 months’ follow-up, i.e. 12 months after the completion of the intervention, sustainably lowering the mean HbA1c in the intervention group to the recommended target below 53 mmol/mol. In addition, clinically modest improvements in total cholesterol and BMI, and an increase in the proportion of patients who achieved the guideline recommended treatment targets for diabetes risk factors were observed in the intervention group compared to controls at 6 months’ follow-up (12).

Based on the observed attendance rate of 87.8 % and high patient satisfaction about the telecoaching programme (97.5 %), good acceptance by patients can be concluded. Qualitative data confirm these findings and show perceived added value by most patients, GPs and all nurses. The COACH Program resulted in improved diabetes understanding and control in the view of most interview participants. Patients associated telecoaching with increased comfort and flexibility, and nurses with efficiency gain. Most providers valued the quality of The COACH Program, particularly the evidence-based advice within the patient progress reports.

Delivering education entirely by phone was perceived as a barrier by nurses. Nurses were convinced that at least one face-to-face contact was necessary at the beginning of the program and expected such a contact to be informative and improve patient trust and commitment. At the same time, the nurses thought that patient education by phone is possible and would help solve the current problem of underfinancing.

Based on the context analysis, most Belgian GPs seem to have grown to trust the competence of diabetes educators in recent years. However, the cooperation between GPs and educators may need further improvement, particularly in the perception of educators who sometimes experience GPs’ reluctance in the area of patient referrals or sharing patient information. The reasons for such reluctance may include increased administrative burden, uncertainty about the programme benefit, fear of losing control or a general limited readiness for innovation, as analysed in previous studies [21, 22]. Effective collaboration between nurses and physicians has the potential to improve quality of care; however, the success factors of such collaboration models need to be further explored [23, 24].

Differences in perceptions between patients and providers, whereby patients show a higher readiness to use telecare, were found in previous research [25]. Providers show more scepticism about the “faceless counselling” and assume lower patient compliance with tele-nurse advice [26, 27]. Provider scepticism about telecare may be caused by limited local evidence of the (cost-) effectiveness and usually project-based financing, altogether explaining a slow uptake of telecare. Lack of a legal, financial and organisational framework for telecounselling was recognised as an important barrier to implementation.

As technological developments will inevitably increase patient demand for mobile and remote services in healthcare, appropriate (regulatory) action by the national healthcare authorities is needed to ensure feasibility and minimal quality standards for such services in chronic care, in the first place where their effectiveness has been proved. A framework to support the necessary changes needs to be developed, including conceptualisation of patient education in multi-morbidity, encompassing the qualification of the educator, the process of providing education and its evaluation, and the scope of interaction with the care team; as well as legal clarity on information security and privacy, professional liability and remuneration of providers’ performance. Lack of a global implementation plan that includes consultations with the stakeholders and the introduction of appropriate organisational and financial measures are believed to limit the adoption of telecare solutions [28, 29].

Overall, there is an unmet need for patient self-management support in diabetes and other chronic conditions, both in primary and in hospital care. It has been reported that about 55 % of patients with cardiovascular diseases in Belgium do not undergo any rehabilitation programme after their discharge from hospital [30], with the main reasons for non-attendance being distance to the hospital, patients’ belief that they can handle their own problems, and lack of time [31]. The evidence to support telecoaching in cardiac rehabilitation has been growing [32, 33] and may offer a solution for some patient groups. Of all the telecoaching programmes in chronic conditions, the COACH Program merits special attention, as in the past 15 years it has proved effective in different chronic conditions, including diabetes and coronary heart disease, and in diverse cultural contexts [12, 13, 34].

Previous implementation research identified several factors influencing the integration of self-management support programmes in daily practice: engagement of general practitioners, patient recruitment methods and quality assurance of the programme delivery [35–37]. Local organisation and financing of primary care also seem to influence the adoption of patient support programmes. While a trend towards strengthening the role of nurses in chronic care delivery has been observed in most European countries, nurse-led approaches remain challenging in systems where primary care is traditionally provided by doctors in solo practices with few support staff [38]. These findings need to be taken into account when working out a local implementation strategy for telecoaching in chronic care.

A combination of qualitative and quantitative research methods in this study allowed for a methodological triangulation [39]. A potential of personal bias was methodologically resolved through parallel coding with subsequent consensus-based interpretation of the interview data. The self-selection and the purposive sampling of the interview respondents may limit the representativeness of the results. The unique nature and an evolving character of the study context and participants may make the findings non-transferable to other settings or to the future [19]. To support future implementation of telecoaching, it may be important to explore multiple patient recruitment techniques and identify those groups of patients who might benefit from diabetes education through alternative delivery modes. With further penetration of the internet, telecoaching can be transferred to internet-based solutions, with the potential to upgrade patient-nurse interaction by adding video-communication, and to improve multidisciplinary team collaboration by creating shared electronic patient records.

## Conclusions

Nurse-led telecoaching of people with type 2 diabetes as part of a clinical trial in Belgium was well-accepted by patients, nurses and GPs, with providers being in general more critical in their reflections. With increasing patient demand for mobile and remote services in healthcare, the findings of this study should support professionals involved in healthcare policy and innovation.

## Additional files

Additional file 1: Interview plan. (DOCX 16 kb)
Additional file 2: Table S1. Barriers and facilitators for implementation of nurse-led telecoaching in type 2 diabetes in Belgian primary care per applied CFIR domain. (DOCX 17 kb)
