# Supplementary material for: Patient and provider acceptance of telecoaching in type 2 diabetes: a mixed-method study embedded in a randomised clinical trial
Source: BMC Med Inform Decis Mak. 2016 Nov 9;16:142. doi: 10.1186/s12911-016-0383-3 (PMC5101679; doi:10.1186/s12911-016-0383-3)
Supplement: Additional file 1: — Interview plan. (DOCX 16 kb) [file 12911_2016_383_MOESM1_ESM.docx]

# Additional file 1

# Interview Plan

**Introduction**

| During the interview, I would like to discuss the following topics: the challenges in diabetes management (diabetes care for providers) that you experience; role of diabetes self-management education, and your experiences with the COACH Program – the programma of diabetes education by phone.  Several GPs, nurses and patients involved in the project, will be interviewed, in order to generalize the views of these different groups. Prior to the beginning, would you, please, sign the informed consent where the research purpose and the data analysis methods guaranteeing confidentiality are explained.  Wouldn’t you object if I would audio-tape the interview? |
| --- |

1. **Interview with GPs**

| **Main topics (T)/ questions (Q)** | **Possible subtopics** |
| --- | --- |
| T1. Diabetes treatment in primary care. Perceived barriers and facilitators.  *Q1. We would like to evaluate the COACH Program within the broad context of care organization in Flanders. What are the challenges and the facilitators of good quality care in you daily practice?*  *Q2. An important initiative was introduction of the care trajectories in type 2 diabetes. What did/ does this initiative mean for your work?* | Knowledge of guidelines  Use of quality indicators  Patient compliance  Patient education  Shared care  Electronic data collection  Financial incentives… |
| T2. Delivery of diabetes education in primary care.  Perceived barriers and facilitators.  *Q. What are your experiences with diabetes education as part of shared care in diabetes? The questions is referred to the usual education and not the COACH Program.* | Contact with educator  Contact with patient  The referral procedure  Education content  Education mode  Education frequency  Reporting  Measuring effect… |
| T3. Experiences with the COACH Program. Perceived benefits and disadvantages.  *Q. How did you experiences the participation of your patient in the COACH Program?*  *Q. What worked for you and what did not?*  *Q. How would you improve the program?* | Information about the program  Contact with coach  Changes in patient behavior  Coaching reports (content and mode of communication)  Contact with the patients  Advice on adjustment of the medication therapy |
| T4. Potential of telecare/ telemedicine in Belgium. Perceived benefits and disadvantages; barriers and facilitators  *Q. What is your opinion about telecare in general?*  Q. (optional) *What factors are important for the implementation of telecare?* | Applications  Pathologies  Legal issues  Ethical issues  Compliance  Willingness to use |

1. **Interview with nurse-educators**

| **Main topics (T)/ questions (Q)** | **Possible subtopics** |
| --- | --- |
| T1. Delivery of diabetes education in primary care.  Perceived barriers and facilitators.  *Q. What are your experiences with your work as diabetes educator (outside of the COACH Program)?* | Knowledge background  Training  Contact with patient  Contact with the care team  The referral procedure  Education content  Education mode  Education frequency  Reporting  Measuring effect  Patient compliance… |
| T2. Experiences with the COACH Program.  Perceived benefits and disadvantages  *Q. How did you experiences working with the COACH Program?*  *Q. What worked for you and what did not?*  *Q. How would you improve the program?* | Training  Support  Combination with other tasks  Software  Contact with GP  Changes in patient behavior  Coaching reports (content and mode of communication)  Contact with the patients  Patient compliance  Advice on adjustment of the medication therapy |
| T3. Potential of telecare/ telemedicine in Belgium. Perceived benefits and disadvantages;  barriers and facilitators.  *Q. What is your opinion about telecare in general?*  *Q. (optional) What factors are important for the implementation of telecare?* | Applications  Pathologies  Safety  Legal issues  Ethical issues  Compliance  Willingness to use |

1. **Interview with patients**

| **Main topics (T)/ questions (Q)** | **Possible subtopics** |
| --- | --- |
| T1. Diabetes self-management. Perceived barriers and facilitators.    *Q. How do you experience your daily life with diabetes?* | Understanding of diabetes  Understanding of treatment goals  Treatment compliance  Communication with GP/ educator/ specialist  Social and emotional coping  Financial concerns… |
| T2. Experiences with the COACH Program. Perceived benefits and disadvantages  *Q. How did you experience your participation in the COACH Program?*  *Q. What worked for you and what did not?*  *Q. How would you improve the program?* | Information about the program  Contact with coach  Program content  Mode of communication  Contact frequency and program duration  Coaching reports  Advice on adjustment of the medication therapy  Changes in knowledge/ behavior (during and after the program) |
| T3. Potential of telecare/ telemedicine in Belgium. Perceived benefits and disadvantages; barriers and facilitators.  *Q. What is your opinion about telecare in general?*  *Q. (optional) What factors are important for the implementation of telecare?* | Legal issues  Ethical issues  Safety  Compliance  Willingness to use |
