# Supplementary material for: Patient and provider acceptance of telecoaching in type 2 diabetes: a mixed-method study embedded in a randomised clinical trial
Source: BMC Med Inform Decis Mak. 2016 Nov 9;16:142. doi: 10.1186/s12911-016-0383-3 (PMC5101679; doi:10.1186/s12911-016-0383-3)
Supplement: Additional file 2: Table S1. — Barriers and facilitators for implementation of nurse-led telecoaching in type 2 diabetes in Belgian primary care per applied CFIR domain. (DOCX 17 kb) [file 12911_2016_383_MOESM2_ESM.docx]

**Additional file 2**

**Table S1:** Themes associated with the barriers and facilitators of The COACH Program adoption in Belgium. Summary of findings.

| **Factors potentially influencing adoption** | **Facilitators** | **Barriers** |
| --- | --- | --- |
| **Intervention characteristics**  Benefit for patient    Key features:  *Communication by phone*    *Structured risk factor management*    *Interaction between coach and GP*  *Advice on medication therapy*    *Use of software for patient administration* | Improved understanding, motivation, risk factor control, discipline in the diet habits, regular check-ups, physical activity (All).  Self-monitoring of blood glucose (P/C).  Regular repetition and control by coach as a vehicle for lifestyle change (P).  Time saving, no transport costs, flexibility in making an appointment, comfort (C/P)  Comprehensive patient information at the start of the program; guidelines-based training in individual risk factor targets and medication management (C)  Trust in the quality of the advice (GP)  Sharing patient progress reports with GP (GP)  Readiness of GPs for cooperation and information exchange with coaches (C)  Trust of GPs in the competences of the coaches (GP)  Quicker feedback to patient between the GP visits (C)  Supporting the session structure; capturing patient progress; helping in preparing a report (C) | Poor patient motivation and therapy compliance (GP/C)  Patient fading perceptive capabilities (C/GP)  Uncertainty about the program benefit (GP)  Estimating the reaction of patient and capturing attention; gaining trust (C).  Commitment for keeping the appointment (C/P).  Remembering and understanding targets for all risk factors (P/C)  Lack of personal contact and immediate interaction on the appropriate therapy adjustment (GP/C)  Paper reports not suitable for integration into the patient electronic file (GP)  Uncertainty of coaches about the acceptance by GPs (C)  Negative perception of GPs about “taking over” their responsibilities (GP)  Reluctance of GPs in medication initiation/uptitration (C)  Postponing attitude of patients in visiting the GP (C)  New way of working; time consuming; occurring technical issues (C) |
| **Implementation Process**  *Engaging of GPs & Patient recruitment*  *Executing & Quality assurance* | Clear communication about the start and the goals of the program (P/GP)  Patient readiness/willingness to make his own choice on participation (P)  Ongoing support and feedback;  Interaction with other coaches;  Receiving a follow-up of patient lab results (C) | Uncertainty about the source, goals and quality of the program (GP)  Poor patient motivation (P)  Telephone-aversion (P)  Combination with other tasks;  Time scarcity;  Patient failure to keep the appointment (C) |
| **Broader context**  *Current practice of patient education*  *in type 2 diabetes*    *Personal beliefs about tele-consultations* | Importance of diabetes education from the moment of the diagnosis (All)  Reimbursement inequalities (All)  Need of a variety of programs tailored to patient needs (GP/C)  Facilitating the work of GPs (GP)  High transport costs to home care organization (C)  No standard curriculum in training of diabetes educators (C)  Efficiency gain and better accessibility through partial substitution of face-to-face contact (All)  Effectiveness in prevention services and monitoring of vital parameters (GP/C) | Patient poor motivation and therapy compliance (GP/C)  Limited availability of diabetes educators (GP)  Lack of GPs’ readiness for cooperation and information exchange (C);  GP’s selective referral behaviour;  Fee-for-service payments of GPs (poor fit with chronic care)  Administrative burden (GP/C)  Uncertainty about the evidence  Inclusion of older patients is difficult  Lack of legal, financial and organizational framework (GP/C) |

C=coaches; GP=General practitioners; P=patients
